# Impact of age on outcome after colorectal cancer surgery in the elderly - a developing country perspective

**DOI:** 10.1186/1471-2482-11-17

**Published:** 2011-08-17

**Authors:** Muhammad Rizwan Khan, Hassaan Bari, Syed Nabeel Zafar, Syed Ahsan Raza

**Affiliations:** 1 Department of Surgery, Aga Khan University & Hospital, Stadium Road, Karachi - 74800, Pakistan

## Abstract

**Background:**

Colorectal cancer (CRC) is a major source of morbidity and mortality in the elderly population and surgery is often the only definitive management option. The suitability of surgical candidates based on age alone has traditionally been a source of controversy. Surgical resection may be considered detrimental in the elderly solely on the basis of advanced age. Based on recent evidence suggesting that age alone is not a predictor of outcomes, Western societies are increasingly performing definitive procedures on the elderly. Such evidence is not available from our region. We aimed to determine whether age has an independent effect on complications after surgery for colorectal cancer in our population.

**Methods:**

A retrospective review of all patients who underwent surgery for pathologically confirmed colorectal cancer at Aga Khan University Hospital, Karachi between January 1999 and December 2008 was conducted. Using a cut-off of 70 years, patients were divided into two groups. Patient demographics, tumor characteristics and postoperative complications and 30-day mortality were compared. Multivariate logistic regression analysis was performed with clinically relevant variables to determine whether age had an independent and significant association with the outcome.

**Results:**

A total of 271 files were reviewed, of which 56 belonged to elderly patients (≥ 70 years). The gender ratio was equal in both groups. Elderly patients had a significantly higher comorbidity status, Charlson score and American society of anesthesiologists (ASA) class (all p < 0.001). Upon multivariate analysis, factors associated with more complications were ASA status (95% CI = 1.30-6.25), preoperative perforation (95% CI = 1.94-48.0) and rectal tumors (95% CI = 1.21-5.34). Old age was significantly associated with systemic complications upon univariate analysis (p = 0.05), however, this association vanished upon multivariate analysis (p = 0.36).

**Conclusion:**

Older patients have more co-morbid conditions and higher ASA scores, but increasing age itself is not independently associated with complications after surgery for CRC. Therefore patient selection should focus on the clinical status and ASA class of the patient rather than age.

## Background

Age has received increasing multidisciplinary attention as a prognostic factor for postoperative complications. Older age is associated with increasing co-morbid conditions, and suitability of surgical candidates based on age has traditionally been debated. The impact of age on postoperative outcome after major colorectal surgery (CRC) remains controversial. Age is a major risk factor for the development of cancer and the incidence of carcinomas increases with advancing age [1]. The incidence of carcinoma of the colon and rectum peaks in the seventh and eight decades of life, with only 5% recorded in those younger than 40 years [2].

Older patients usually present with coexisting diseases and whether these patients are capable of enduring extensive gut resection or not, is a major decision the surgeon has to make. As a result, surgery for the treatment of CRC has been influenced by age [3]. Previous studies have shown that the rates of emergency presentation, inoperability and peri-operative mortality were high in elderly patients with colorectal cancer [4]. Such data might lead to withholding curative treatment with radical surgical procedures and opting for more "conservative" or palliative therapies in elderly patients. But many recent publications have encouraged the same surgical approach as for younger patients [5-7].

The risks and benefits of surgery for CRC in old patients have not yet been clearly defined in Pakistan. Due to lack of local data elderly patients may be refused life saving surgery, with the assumption that the outcome will be poor; making surgery futile. The aim of this study was to evaluate the impact of age on colorectal cancer presentation, surgical management and early postoperative outcomes from a single institution of a developing country and to determine whether old age itself is a predictor of complications after colorectal cancer surgery.

## Methods

Patients with pathologically confirmed Colorectal Cancer who underwent primary surgery at the Aga Khan University Hospital (AKUH) Karachi, Pakistan, between January 1999 and December 2008 were identified from medical records and their files were reviewed. AKUH is one of the largest private tertiary care hospitals in Pakistan. With 563 beds it provides services to over 50,000 hospitalized patients and to over 600,000 outpatients annually. Despite the lack of a specific referral pattern in the country, a diverse group of patients from all over Pakistan seek healthcare at AKUH.

Identified patients were divided into two groups on the basis of chronological age: ≥ 70 years (Group I, elderly patients) and < 70 years (Group II). There is lack of a consistent definition of old age in the literature. Authors have used a number of different cut offs ranging from 65 to 85 years in various studies [8-14]. According to the Ministry of Population Welfare of the Government of Pakistan, average life expectancy in Pakistan is 66.4 years [15]. Due to genetic and environmental factors South Asian's generally age faster than most western societies. In Pakistan a person is usually physically unable to cope with work by the age of 70. Based on these facts we used 70 years as threshold in our study. However we also performed sensitivity analysis using 65 and 60 years as a cut off.

Clinical and pathologic characteristics of these cases were recorded with the help of a structured questionnaire including: patients demographics, associated co-morbids, ASA (American Society of Anesthesiologist) levels, albumin levels for nutritional status, preoperative complications, mode of admission, site of tumor, TNM staging, number of lymph nodes positive for tumor metastasis, early postoperative complications and 30 day mortality. Based on the co-morbid conditions, Charlson's index was calculated for each patient [13]. Early post-operative morbidity was defined as complications developing within 30 days after the operation. They were classified into surgical (wound infection, anastomotic leak, abdominal sepsis/abscess, paralytic ileus and intestinal obstruction) and systemic complications (postoperative urinary tract infection, difficulty in voiding, pneumonia, pleural effusion, myocardial infarction, atrial fibrillation, systemic sepsis and stroke).

Data was analyzed using Statistical Package for Social Sciences (SPSS version 16.0). Descriptive statistics were computed for characteristics of patients, laboratory, tumor and postoperative morbidity and mortality. Fisher's exact test was used to compare patients above and below 70 years of age. Occurrences of early postoperative systemic and surgical complications were also compared between these groups. Multivariate logistic regression analysis was performed separately for systemic and surgical complications. We were unable to use mortality in a multivariate model owing to the small number of events (deaths = 6). Variables selected for multivariate analysis were age and an a priori list of clinically relevant variables.

This research project was a retrospective review of medical records and in accordance with the ethical principles laid forth by the Declaration of Helsinki, retrospective reviews of medical records in which patient identifying information is not collected are exempt from formal ethical review by the ethical review committee of the Aga Khan University, the institution where the research was performed [16]. Being a review of 10 years of data consent from individual patients was not sought. No identifying information was recorded by the research team. All information was collected from the medical records and no questionnaire was administered to the patient.

## Results

A total of 271 patients underwent surgical treatment for pathologically confirmed primary colorectal cancer during the study period, out of which 56 patients were ≥ 70 years (Group I) and 215 were < 70 years old (Group II) at the time of surgery. An overall 16 surgeons performed surgeries over the 10 year study period. All of these were experienced consultants and we found no difference in the proportion of surgeries performed by surgeons in each group (p = 0.4)

In Group I, 73.2% patients were male and the mean age was 75.6 years (range 70 to 88 years); whereas in Group II, 63.3% patients were male and the mean age was 50.1 years (range 15 - 69 years) (table 1). The most common site of tumor in elderly group was sigmoid colon (41%) followed by rectum (26.8%), compared to Group II where rectum (37.7%) was the most frequent site of tumor and sigmoid colon (16.7%) was second to it. Adenocarcinoma was the most frequent type of tumor (Group I: 100%, Group II: 97.2%) and majority of the tumors were moderately differentiated (Group I: 80.4%, Group II: 65.1%). Stage IV tumors were more frequently noticed in Group I (7.1%) compared to Group II (4.7%). All these differences however, were non-significant between the two groups.

**Table 1:** Presentation of colorectal cancer by age categories

Variables	Age ≥ 70 N = 56 n(%)	Age < 70 N = 215 n(%)	P value
			
Mean age, yr (range)	75.6 (70 - 88)	50.1 (15 - 69)	< 0.001
Gender			0.207
Male	41 (73.2)	136 (63.3)	
Female	15 (26.8)	79 (36.7)	
Site of Tumor			0.207
Rectum	15 (26.8)	81 (37.7)	
Colon			
Sigmoid	23 (41)	36 (16.7)	
Descending	1 (1.8)	17 (7.9)	
Left angle	1 (1.8)	7 (3.3)	
Transverse	1 (1.8)	8 (3.7)	
Right angle	0	8 (3.7)	
Ascending	15 (26.8)	58 (27)	
Type of Tumor			-
Adenocarcinoma	56 (100)	209 (97.2)	
Squamous	0	3 (1.4)	
Melanoma	0	3 (1.4)	
Grade of Tumor			0.061
Well differentiated	8 (15.4)	36 (16.7)	
Moderately differentiated	45 (80.4)	140 (65.1)	
Poorly differentiated	2 (3.6)	33 (15.3)	
Stage of Tumor			0.262
I	15 (26.8)	26 (12.1)	
II	15 (26.8)	76 (35.3)	
III	20 (35.8)	96 (44.7)	
IV	4 (7.1)	10 (4.7)	
Not determinable	2 (3.5)	7 (3.2)	

No significant difference was noticed in the rate of overall preoperative complications (Group I: 83.9%, Group II: 86%, *p *= 0.68), the commonest complication being bowel obstruction (Group I: 17.9%, Group II: 11.6%, *p *= 0.21) (table 2). Rate of emergency presentation was also nearly similar (Group I: 17.9%, Group II: 12.1%, *p *= 0.25). Although surgeries with palliative intent were more frequently performed in elderly patients, the difference was not significant (Group I: 7.1%, Group II: 4.7%, *p *= 0.45); The most commonly performed surgeries were right hemicolectomy (26%), abdominoperenial resection (21%), sigmoid colectomy (6%) and low anterior resections (12%). Only 5 laparoscopic procedures were performed (Group I: 4 (2%), Group II: 1 (2%), *p *= 0.88). The rate of hypoalbuminemia was insignificantly high in Group I (*p*= 0.80). Presence of co-morbid conditions (Group I: 82.1%, Group II: 45.1%, *p*< 0.001), higher ASA levels (Group I: 53.6%, Group II: 28.4%, *p*< 0.001) and Charlson score of 3 and above (Group I: 96.4%, Group II: 11.6%, *p*< 0.001) were significantly more frequently recorded amongst elderly patients when compared to patients who were < 70 years old.

**Table 2:** Comparison of pre-operative, operative and post operative variables between the two age groups in patients undergoing surgery for colorectal cancer

Variables	Age ≥ 70 N = 56 n(%)	Age < 70 N = 215 n(%)	*p*
**Pre-operative/operative**			
Hypoalbuminemia	17 (30.4)	47 (21.9)	0.80
Preoperative complications	47 (83.9)	185 (86)	0.68
Obstruction	10 (17.9)	25 (11.6)	0.21
Perforation	2 (3.6)	7 (3.3)	0.90
Co-morbidity	46 (82.1)	97 (45.1)	< 0.001
ASA 3-5	30 (53.6)	61 (28.4)	< 0.001
Charlson score (3 & above)	37 (67.3)	52 (24.1)	< 0.001
Mode of presentation			0.25
Emergency	10 (17.9)	26 (12.1)	
Elective	46 (82.1)	189 (87.9)	
Intent of Surgery			0.45
Curative	52 (92.9)	205 (95.3)	
Palliative	4 (7.1)	10 (4.7)	
Type of Surgery			0.15
Right hemicolectomy	16 (28.6)	55 (25.6)	
Abdominoperenial resection	5 (8.9)	52 (24.1)	
Low anterior resection	9 (16.1)	23 (10.7)	
Sigmoid Colectomy	9 (16.1)	26(12.1)	
Others	17 (30.2)	59 (27.4)	
**Post-operative**			
Overall morbidity	23 (41.1)	75 (34.9)	0.39
Systemic complications	16 (28.6)	37 (17.2)	0.05
Surgical complications	11 (19.6)	54 (25.1)	0.39
30 d mortality	4 (7.1)	2 (0.9)	0.005

Postoperative complications occurred in 36.2% (n = 98) patients. In Group I, 41.1% patients developed postoperative complications compared to 34.9% of Group II (*p *= 0.39) (table 2). Upon cross tabulation, there was no significant difference in the development of postoperative surgical complications between the two groups (*p*= 0.39); however, systemic postoperative compilations differed significantly (Group I: 28.6%, Group II: 17.2%, *p*= 0.05). The postoperative 30-day mortality rate was 7.1% in Group I compared to 0.9% in Group II (*p*< 0.001).

The difference in systemic complications by age disappeared upon multivariate analysis (table 3). Age was not associated with either systemic or surgical complications after adjusting for other factors (95% CIs = 0.32-1.66 and 0.63-3.62). Factors that significantly predicted higher systemic complications were tumor site, higher ASA score and pre-operative bowel perforation. Tumors in the rectum were 2.5 times more likely to have complications as compared to those in the colon (95% CI = 1.21-5.34). Patients with an ASA score of > 2 were 2.9 times more likely to have systemic complications than those with lesser scores (95% CI = 1.30-6.25). Pre-operative perforation was associated with a higher rate of both systemic and surgical complications with high odds ratios. Patients presenting with perforation were 9.7 times (95% CI = 1.94-48.0) and 8.3 times (95% CI = 1.72-39.7) more likely to have systemic and surgical complications respectively. Similar results were found when ages of 65 and 60 were used as cut offs to form the two groups. Age remained an insignificant covariate in the multivariate models.

**Table 3:** Multivariate analysis of factors associated with post-operative complications in patients undergoing surgery for colorectal cancer

Variable	Systemic complications		Surgical complications	
	OR	95% CI	OR	95% CI
Age < 70 years	0.73	0.32 - 1.66	1.51	0.63 - 3.62
Gender- female	0.66	0.31 - 1.40	0.72	0.37 - 1.40
Tumor site- Rectum	2.54*	1.21 - 5.34	1.01	0.52 - 1.95
High tumor grade	1.60	0.58 - 4.43	2.54	0.94 - 6.87
High tumor stage	1.21	0.61 - 2.39	1.22	0.66 - 2.29
High ASA score	2.86*	1.30 - 6.25	1.84	0.84 - 4.03
High Charlson score	1.17	0.53 - 2.59	0.79	0.36 - 1.70
Pre-Op perforation	9.65*	1.94 - 48.0	8.27*	1.72 - 39.7
Palliative surgery	1.19	0.15 - 9.26	0.83	0.19 - 3.63
Elective surgery	0.62	0.21 - 1.83	0.45	0.17 - 1.14

* Significant at 5% significance level

## Discussion

A number of geriatric patients develop colorectal cancer, which is a major cause of morbidity and mortality in this age group. Surgery, whether palliative or curative, is the treatment of choice for majority of patients with colorectal cancers. In the recent past, extensive surgical procedures in elderly patients seemed to be contraindicated because of poor functional status, associated co-morbids and impaired cognition [17]. However improved diagnostic procedures, intensive peri-operative care, better anesthesia and surgical techniques have made it possible to perform high risk surgical procedures in old age patients [18].

Our study demonstrates that age alone is not a predictor of postoperative complications in a Pakistani population of patients with colorectal cancer. In a previous study we describe rates of complications and their management at our institution in greater detail [19]. A number of Western studies also demonstrate that age is not a predictor of post operative complications [5-7,10-13,20]; however, to our knowledge this is the first study to demonstrate these results in our region. Albeit the limitations of this study, it seems that even in our population colorectal surgery should not be deferred solely on the basis of age. Elderly patients have similar rates of morbidity and mortality as younger patients of the same clinical status.

In our study postoperative surgical complication rates were nearly similar in both age groups. There was however a higher rate of postoperative systemic complications upon univariate analysis. Twenty eight percent of patients of age ≥ 70 years developed systemic complications after CRC surgery in our study which is comparable to previous studies [4,21,22]. An adjusted model clearly demonstrated that any effect of age on systemic complications was explained by other factors such as ASA score of the patient.

Elderly patients usually have a higher risk profile [22-24]. This was also found in our analysis. Almost 82% of all patients in the ≥ 70 years old group had some co-morbid compared to 45% of younger age group (p < 0.001). This led to significantly higher ASA classification and more than 50% patients of ≥ 70 years old had an ASA score of 3 or above compared to 28.4% patients of < 70 year old group. Similarly significant differences were noticed in Charlson score between the two age groups. A number of studies have demonstrated an association of ASA class with early postoperative morbidity and mortality rates in patients undergoing surgery for CRC [20,25,26]. We found systemic complications to be three times more likely to occur in patients with an ASA of 3 or more.

Postoperative mortality rate was 7.1% in the elderly group compared with 0.9% in those under 70 years old (p = 0.005). This significant increase in mortality rate in elderly is comparable to previous studies [6,7,11,27,28]. This higher mortality rate is very likely also due to other factors such as comorbids or pre-operative clinical condition. Five out of the 6 deaths occurred in our patients with an ASA class of > 2. Due to the small number of deaths (n = 6), we were unable to perform multivariate analysis with mortality as an outcome which would have demonstrated this clearly.

There is an increasing trend in Western societies towards more curative treatment for elderly patients with colorectal cancer [18]. However this practice is unclear in developing countries and many elderly patients with CRC in countries like Pakistan still receive inadequate treatment solely based on their age. A number of predictive tools are available that assess risk of morbidity and mortality after surgery based on the comorbid and general health status of the elderly [29,30]. It would be beneficial to test these tools on local populations and use these rather than age alone as criteria to select surgical treatment.

This study was from a single institution and even though it is from a large number of patients with representation from all ethnic groups found in Pakistan, the generalizability may be limited. Further studies from other institutions are needed to substantiate our findings. The number of about 250 - 300 patients may seem less in comparison to Western studies, however health seeking behavior in our population differs. There is no screening program for CRC thus many such patients are never diagnosed nor treated appropriately. In addition AKUH is a private institution where patients pay out of their pocket to receive treatment which may account for the fewer patients. The number of patients in the elderly group is low at 56, this is not surprising as life expectancy in Pakistan is low at 65 and not many elderly patients seek surgical treatment. The study does however provide valuable evidence that could be used by surgeons in decision making and patient selection. Since the purpose of this study was to determine the independent effect of age on post operative complications, using a cut off of 70 years may be considered inadequate by some. As a secondary exercise we did perform the same analysis using different cut offs of age; 60 and 65 years. The results were the virtually same each time. The purpose of this study was to determine the effect (or lack of effect) of age alone and thus other significant variables in the final model were not explored or discussed. These could possibly be areas for further research.

## Conclusions

Age itself is not a risk factor for the development of complications in patients undergoing surgery for colorectal cancer. Age alone should not be a reason to avoid therapeutic or palliative surgery in these patients, instead patient selection should focus on clinical condition and ASA levels.

## Competing interests

The authors declare that they have no competing interests.

## Authors' contributions

MRK was involved in the concept, design and overall supervision of the study. SNZ and SAR were involved data analysis and interpretation. HB and SNZ were involved in manuscript writing. HB retrieved data from medical records. All authors have reviewed and approved of the final manuscript.

## Pre-publication history

The pre-publication history for this paper can be accessed here:

http://www.biomedcentral.com/1471-2482/11/17/prepub
